# Immune evasion, infectivity, and membrane fusion of SARS-CoV-2 variants LP.8.1.1, XEC.25.1, XFG, and NB.1.8.1

**DOI:** 10.1128/spectrum.03826-25

**Published:** 2026-06-24

**Authors:** Feng Jiang, Miao Zheng, Long Gao, Yanhang Zhuo, Xiaoqin Liang, Zhiwei Chen, Xinghua Huang, Yisheng Chen, Zhaonan Zeng, Yufan Xiao, Xiaohong Du, Frank Xiao‐Feng Qin, Weihua Liu, Haijun Tang

**Affiliations:** 1 Shengli Clinical Medical College, Fujian Medical University 74551, Fuzhou, Fujian, China; 2 Center for Experimental Research in Clinical Medicine, Fujian Provincial Hospital 117861, Fuzhou, Fujian, China; 3 Fuzhou University Affiliated Provincial Hospital 117861, Fuzhou, Fujian, China; 4 Department of Pain Management and Vascular Surgery & Interventional Oncology, Fujian Provincial Hospital 117861, Fuzhou, Fujian, China; 5 College of Integrative Medicine, Fujian University of Traditional Chinese Medicine 47858, Fuzhou, Fujian, China; 6 Department of Infectious Disease, the First Affiliated Hospital of Anhui Medical University 36639, Hefei, Anhui, China; 7 Fuzhou Center for Disease Control and Prevention 544219 , Fuzhou, Fujian, China; 8 Department of Preventive Medicine, School of Public Health, Fujian Medical University 74551 , Fuzhou, Fujian, China; 9 Department of Gastroenterology, Fujian Provincial Hospital 117861, Fuzhou, Fujian, China; 10 National Key Laboratory of Immunity and Inflammation, Suzhou Institute of Systems Medicine, Chinese Academy of Medical Sciences & Peking Union Medical College 570570, Suzhou, Jiangsu, China; 11 Key Laboratory of Synthetic Biology Regulatory Elements, Suzhou Institute of Systems Medicine, Chinese Academy of Medical Sciences & Peking Union Medical College 570570, Suzhou, Jiangsu, China; 12 Department of Nephrology, Shengli Clinical Medical College of Fujian Medical University, Fujian Provincial Hospital, Fuzhou University Affiliated Provincial Hospital 117861, Fuzhou, Fujian, China; Shandong First Medical University, Jinan, Shandong, China

**Keywords:** JN.1 subvariants, immune escape, infectivity, membrane fusion

## Abstract

Emerging SARS-CoV-2 variants, with potentially enhanced immune evasion and transmissibility, pose a serious challenge to public health. This study provides a systematic characterization of the virological features of JN.1 descendant subvariants, including LP.8.1.1, KP.3, XEC.25.1, XFG, and NB.1.8.1, focusing on their infectivity, receptor binding, membrane fusion, and immune evasion capacity. Multiple JN.1 descendant variants exhibited reduced infectivity across various human cell lines, whereas XEC.25.1 and NB.1.8.1 demonstrated significantly enhanced cellular infectivity. JN.1 descendant variants retained the capability to infect cells expressing ACE2 orthologs from diverse mammalian species, indicating their persistent risk of zoonotic transmission. Notably, NB.1.8.1 exhibited the highest ACE2-binding affinity among all tested variants, while XEC.25.1 shows superior membrane fusion activity, particularly in cells expressing TMPRSS2. Serum neutralization assays revealed that LP.8.1.1, KP.3, XEC.25.1, XFG, and NB.1.8.1 exhibited enhanced immune evasion capabilities compared to the JN.1 strain, which was closely associated with their rapid transmission. These findings reveal that emerging JN.1 subvariants accelerate viral transmission by altering receptor-binding affinity, optimizing cellular entry efficiency, and enhancing immune evasion. Therefore, it is necessary to update vaccines and improve antiviral treatment strategies to meet the public health challenges posed by these variants.

**IMPORTANCE:**

SARS-CoV-2 JN.1 has continuously evolved during the epidemic, giving rise to multiple descendant variants. Currently, JN.1 sublineages NB.1.8.1, XFG, XEC.25.1, and LP.8.1.1 have emerged as the predominant circulating variants globally. The cellular infectivity, cross-species transmission potential, and immune evasion capacity of these emerging variants remain poorly characterized. This study employed a VSV pseudovirus system to characterize the virological features of JN.1 descendant subvariants. We found that the cellular infectivity of JN.1 descendant variants was significantly altered, which may be attributed to changes in receptor-binding affinity or membrane fusion activity. The emerging JN.1 subvariants retained the ability to infect cells expressing ACE2 orthologs from diverse species. Furthermore, the emerging variants LP.8.1.1, KP.3, XEC.25.1, XFG, and NB.1.8.1 exhibited enhanced immune evasion capabilities compared to the JN.1 strain. Our study underscores the importance of surveillance and virological research for emerging JN.1 descendant variants.

## INTRODUCTION

The continuous evolution of SARS-CoV-2 has led to the emergence of new variants, posing a significant threat to public health (1–3). Following the epidemic of the JN.1 strain, SARS-CoV-2 has further evolved into new subvariants, accelerating its global dissemination. Currently, SARS-CoV-2 JN.1 descendant lineages, including NB.1.8.1, XFG, XEC.25.1, and LP.8.1.1, have emerged as the dominant circulating variants globally (4, 5). The NB.1.8.1 sublineage originated from XDV and was characterized by Q493E and A435S mutations, which has been circulating in many countries (4, 5). The XFG variant, generated by recombination between LF.7 and LP.8.1.2, contains four key spike mutations (V445R, N487D, Q493E, and T572I) and has spread rapidly since it was first identified in Canada (4). XEC.25.1 is a descendant of the KP.3 strain, carrying the A435S mutation, and shows a significant transmission advantage (4). Similarly, the LP.8.1.1 variant evolved from KP.1.1 and is characterized by key spike protein mutations (V445R, Q493E, N679R), which are associated with enhanced immune evasion and potentially increased transmissibility (4, 6). New mutations in JN.1 descendant variants may enhance viral transmissibility, potentially increasing the risk of reinfection in human populations. Emerging JN.1 sublineages continue to evolve and adapt to their hosts, but their immune escape capacity, infectivity characteristics, cross-species transmission potential, and host interaction mechanisms remain poorly understood.

The infectious capacity of ARS-CoV-2 is determined by a multi-step process. During viral assembly, the spike protein undergoes pre-activation by furin (7). After virus binding to the ACE2 receptor, the spike protein is further proteolytically cleaved by TMPRSS2 on the cell surface or by cathepsins in endosomes, which triggers membrane fusion and mediates virus entry into target cells (7, 8). Genetic variations in SARS-CoV-2 may affect its cellular infectivity. It has been demonstrated that the SARS-CoV-2 D614G mutation, which emerged early in the pandemic, induces a conformational change in the viral spike protein (9). This structural shift results in a greater proportion of receptor-binding domains (RBDs) adopting an open conformation, thereby enhancing the capacity of the spike protein to bind to the ACE2 receptor (9, 10). Emerging variants harbor a substantial number of novel mutations capable of altering receptor affinity or enabling the use of alternative receptors, potentially increasing the risk of transmission. Compared with the previously dominant JN.1 variant, the infectivity of LP.8.1.1, XEC.25.1, XFG, and NB.1.8.1 toward target cells has not been systematically evaluated.

SARS-CoV-2 spikes are the primary target for neutralizing antibodies elicited by viral infection (11, 12). During viral evolution, accumulated mutations in the variant spike may alter viral antigenicity, thereby facilitating immune evasion from neutralizing antibodies (13–15). Notably, the extensive mutations observed in the JN.1 descendant lineages have raised concerns regarding their potential to evade antibody-mediated neutralization. Previous studies have demonstrated that BA.5 breakthrough infection narrows the diversity of neutralizing antibody epitopes and shifts the antibody response toward non-neutralizing types (16). This indicates that Omicron-induced humoral immunity may have limited effectiveness against emerging subvariants. Therefore, it is crucial to assess whether convalescent serum from COVID-19 patients retains neutralizing antibodies effective against JN.1-derived subvariants. This study focuses on recently predominant circulating variants, including LP.8.1.1, XEC.25.1, XFG, and NB.1.8.1. We evaluated the neutralizing activity of convalescent sera from individuals recovered from either BA.5 infection or sequential BA.5 and XBB.1.5 infections against JN.1-derived variants. Meanwhile, we systematically characterized the biological properties of these novel JN.1-derived variants by assessing their cross-species infectivity, ACE2-binding affinity, membrane fusion activity, and viral entry mechanisms.

## MATERIALS AND METHODS

### Serum samples

Serum samples were obtained from individuals who experienced breakthrough infections with BA.5 or BA.5 + XBB.1.5 (1 month) after receiving two doses of inactivated COVID-19 vaccines (Table S1). The inactivated vaccines administered to the serum donors were BBIBP-CorV (vaccine strain: Wuhan-Hu-1/HB02) and/or CoronaVac (vaccine strain: Wuhan-Hu-1/CZ02).

### Monoclonal antibodies

Monoclonal antibodies (mAbs) were synthesized by AtaGenix based on the sequences deposited in the Protein Data Bank. These antibodies, including BD55-5514, S2H97, VIR-7831, Bebtelovimab, LY-CoV555, AbB505, AbB606, AbE450, and AbG106, all target the RBD of the SARS-CoV-2 spike protein. All antibodies were aliquoted and stored at −80°C to avoid inconsistencies caused by repeated freeze-thaw cycles.

### Cell lines

Cells 293T, Vero, Huh7, Caco-2, and A549 were obtained from the American Type Culture Collection. The human colon adenocarcinoma cell line Caco2 was purchased from Pricella. 293T-ACE2, 293T-ACE2-TMPRSS2, Caco2-ACE2, and A549-ACE2 cells were generated via lentivirus-mediated gene transduction.

### Production of pseudoviruses

Plasmids encoding the spikes of different SARS-CoV-2 variants were synthesized by Genscript Biotechnology. SARS-CoV-2 pseudoviruses were generated based on the vesicular stomatitis virus (VSV) backbone, as described in our previous study (17). The pseudovirus particles were produced by replacing the VSV glycoprotein with the SARS-CoV-2 variant spike. Briefly, 293T cells were transfected with plasmids encoding the SARS-CoV-2 spike employing Lipofectamine 3000 (Invitrogen). At 12 h post-transfection, cells were inoculated with G*ΔG-VSV pseudoviruses. After 6 h of inoculation, cells were washed three times and supplemented with complete medium. Following 24 h of incubation, the pseudovirus-containing supernatant was collected and stored at −80°C.

### Pseudovirus infection assay

Prior to the initiation of the pseudovirus infection assay, viral particles were normalized to equivalent quantities by quantitative RT-PCR (qRT-PCR). The day before the viral infection assay, target cells were seeded in 96-well plates. The next day, the medium was discarded and replaced with 100 μL of medium containing pseudovirus. At 24 h post-infection, cells were lysed with passive lysis buffer (Promega). Following the addition of luciferase substrate (Promega) to the cell lysate, luminescence was quantified with a SpectraMax L microplate reader (Molecular Devices).

### Flow cytometry analysis of receptor affinity

The binding affinities of SARS-CoV-2 variant spike proteins to the ACE2 receptor were evaluated by flow cytometry (18). Briefly, 293T cells were transfected with plasmids encoding the variant spike proteins using Lipofectamine 3000. At 36 h post-transfection, cells were dissociated with EDTA and washed twice with 1 mL of staining buffer. To assess binding activity, cells were incubated with soluble recombinant human ACE2-hFc fusion protein (Sino Biological, 1 μg/mL) for 1 h, followed by staining with a PE-conjugated anti-human IgG Fc secondary antibody (BioLegend). The concentration of ACE2-hFc used in the binding experiments was selected based on preliminary dose-response experiments to ensure it fell within the range of binding saturation (Fig. S3A and B). In parallel, to monitor surface expression, cells were incubated with a mouse anti-SARS-CoV-2 spike antibody (GeneTex, 1 μg/mL) for 1 h, followed by incubation with a PE-conjugated anti-mouse IgG secondary antibody (BioLegend). After a final wash, cells were resuspended in staining buffer and analyzed using an Accuri C6 flow cytometer (BD Biosciences, USA). 293T cells transfected with empty plasmids and subjected to the same staining procedures served as negative controls. Receptor-binding activity was quantified by mean fluorescence intensity (MFI) and normalized to the cell surface expression levels of spike proteins (sACE2/S2).

### Effect of inhibitors on the entry of JN.1 subvariants into cells

Prior to the assay, 293T-ACE2 or 293T-ACE2-TMPRSS2 cells were plated in 96-well plates at 3 × 10^4^ cells/well. The next day, target cells were pretreated with various concentrations of inhibitors for 2 h prior to infection with variant pseudoviruses. The efficiency of viral infection was measured by luciferase activity in cell lysates 24 h post-infection.

### Cell-cell fusion assay

To detect cell-cell fusion between the variant spike and its receptor ACE2, we used a T7 polymerase-based reporter gene system. This system contains two vectors, including the T7 promoter-IRES-luc2 reporter unit (T7 pro) and the T7 polymerase-T2A-EGFP expression unit (T7 pol). Donor cells (293T) were transfected with plasmids encoding the variant spike, T7 pro, and pRL-TK, while acceptor cells (293T-ACE2 or 293T-ACE2-TMPRSS2) were transfected with a T7 pol expression plasmid. After 36 h of transfection, the cells were resuspended to a density of 2 × 10^6^/mL, mixed at a donor-recipient ratio of 2:1, and co-cultured at 37°C for 12 to 16 h. Cell syncytium formation was observed under fluorescence microscopy. Subsequently, the cell lysates were subjected to dual luciferase reporter assays (Promega system).

### Pseudovirus neutralization assay

To evaluate the neutralizing activity of the serum samples, they were subjected to serial fivefold dilutions starting at an initial concentration of 1:40. The diluted samples were incubated with 1000 TCID50 pseudovirus for 1 h at 37°C. Next, 293T-ACE2 cells were inoculated with the serum-virus mixture and cultured at 37°C for 24 h. Neutralizing antibody levels in serum samples were quantified via measurement of luciferase activity in cell lysates. Cells inoculated with pseudovirus in the absence of serum served as the control group.

### Statistical analysis

Statistical analyses were performed using GraphPad Prism 8, and the experimental data were expressed as mean ± standard deviation (SD). The half-maximal inhibitory concentration (IC50) and half-maximal inhibitory dilution (ID50) were determined in GraphPad Prism 8 using a four-parameter dose-inhibition response model. For comparisons of viral infectivity, ACE2-binding affinity, cell-cell fusion, and viral entry pathways, a two-tailed Student’s *t*-test with Welch correction was employed. The Wilcoxon paired signed-rank test was used to evaluate serum neutralizing activity against variant pseudoviruses. A *P* value less than 0.05 was considered statistically significant. Significance levels in figures were indicated as follows: “ns” for not significant, *P < 0.05, **P < 0.01, ***P < 0.001, and ****P < 0.0001.

## RESULTS

### SARS-CoV-2 variants XEC.25.1 and NB.1.8.1 exhibit increased infectivity in multiple susceptible cells

**Fig 1:**
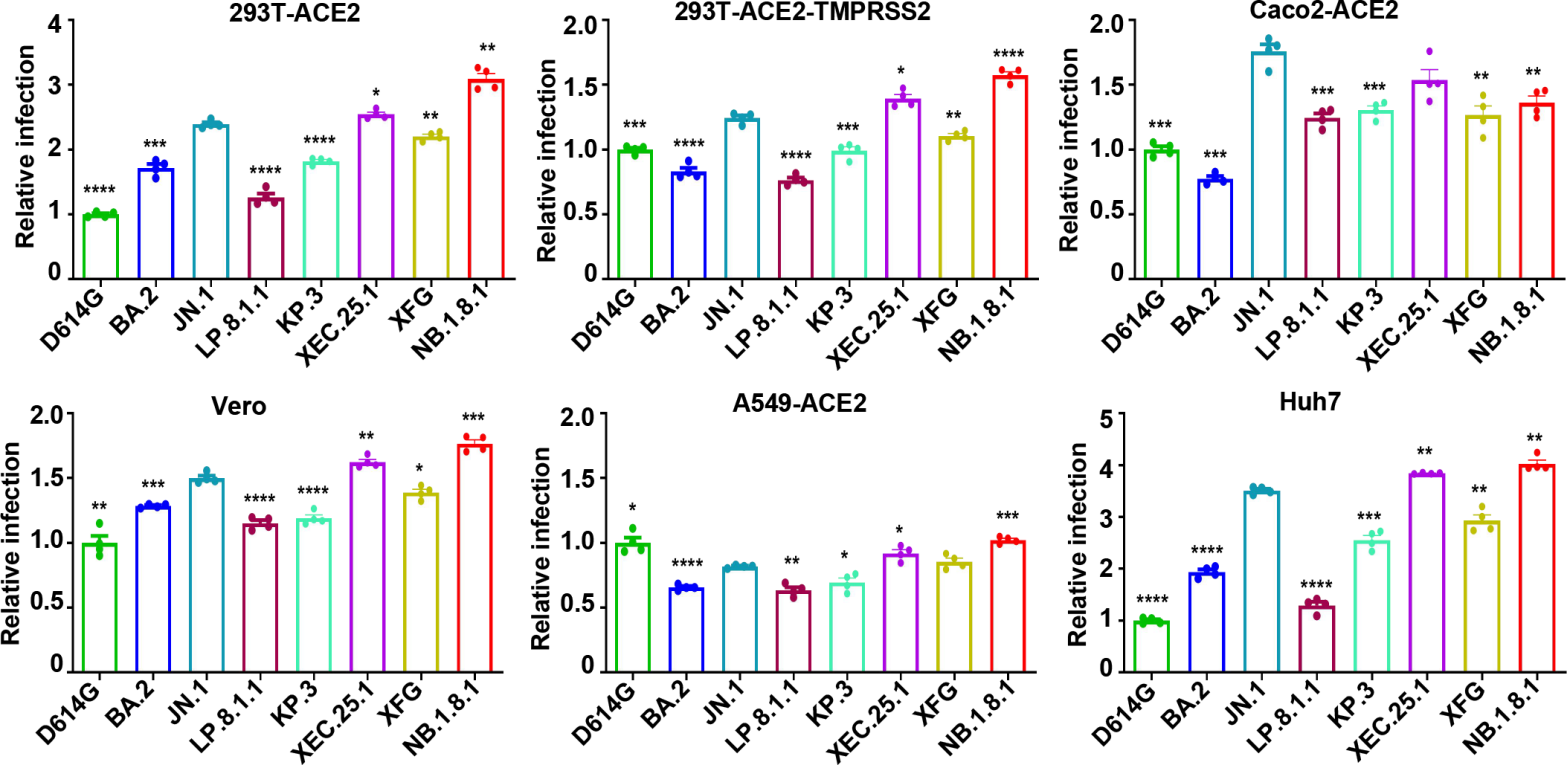
Infection analysis of JN.1-related variant pseudoviruses in susceptible cells. Cells including 293T-ACE2, 293T-ACE2-TMPRSS2, Caco2-ACE2, Vero, A549-ACE2, and Huh7 were infected with pseudoviruses carrying SARS-CoV-2 variant spikes. The JN.1 variant served as the reference strain for all statistical comparisons. Experiments were performed with four replicates and independently repeated at least twice. One representative is shown with error bars indicating SD. Two-tailed Student’s *t*-tests were used for statistical analysis, with *P* values shown as *P  <  0.05, **P  <  0.01, ***P  <  0.001, ****P  <  0.0001.

To investigate the infection characteristics of currently circulating SARS-CoV-2 variants, we generated eight pseudoviruses, including D614G, BA.2, JN.1, LP.8.1.1, KP.3, XEC.25.1, XFG, and NB.1.8.1 (Fig. S1A and B). These pseudoviruses were prepared using the VSV system, and their infection efficiency was evaluated in multiple susceptible cells. As previously reported, the JN.1 variant exhibited higher cellular infectivity than the ancestral D614G and BA.2 strains in most cells (Fig. 1). Pseudoviruses carrying XEC.25.1 and NB.1.8.1 spikes exhibited enhanced infectivity relative to JN.1 in the majority of tested cells, with the exception of Caco2-ACE2. However, pseudoviruses carrying the LP.8.1.1, KP.3, and XFG spikes exhibited reduced infectivity compared to JN.1. Among them, LP.8.1.1 showed the lowest infectivity, with an approximately 1.3- to 2.7-fold reduction.

### The JN.1 descendant lineages exhibit broad tropism toward mammalian species

**Fig 2:**
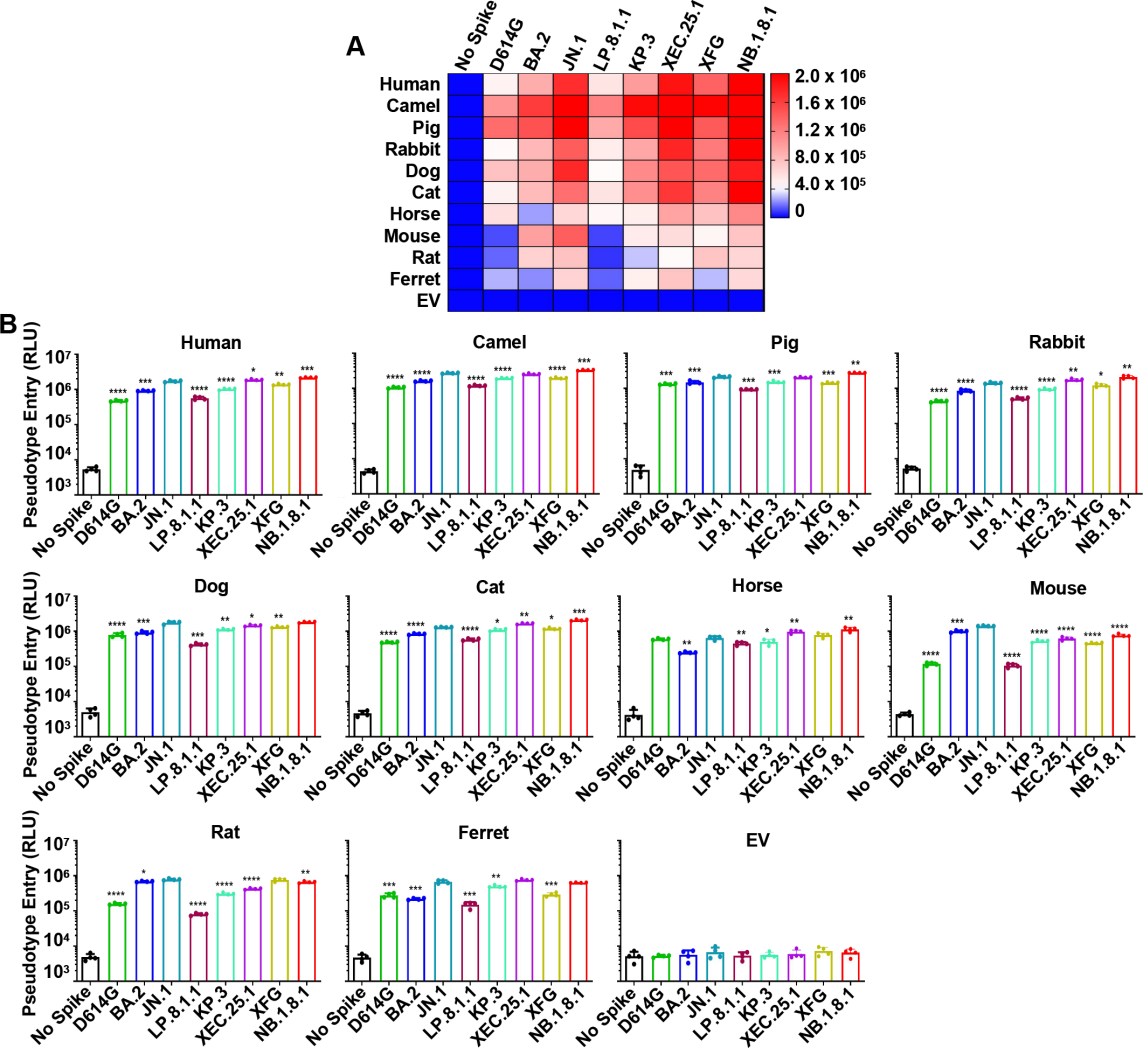
The cross-species infection potential of emerging JN.1-related variants. (**A**) The heat map depicts the cross-species infection performance of JN.1-related variants in 293T cells expressing homologous ACE2 receptors from various species. (**B**) The infective capacity of JN.1-related variants in 293T cells expressing ACE2 from various species. All statistical analyses were conducted in comparison to the JN.1 variant. The experiments were performed with four technical replicates and repeated at least twice. Data from one representative experiment were presented as the mean ± SD. Statistical significance was determined by two-tailed Student’s *t*-tests, with significance indicated by *P < 0.05, **P < 0.01, ***P < 0.001, and ****P < 0.0001. EV represents the empty vector.

SARS-CoV-2 initiates cellular invasion via spike-mediated recognition and binding to host ACE2 receptors. To evaluate the cross-species transmission potential of currently circulating variants, we assessed their infectivity across cells expressing ACE2 receptors from 10 distinct species. These species are closely associated with human life, and some of them have been identified as hosts or intermediate hosts for various zoonotic viruses, which may increase the potential risk of viral transmission across species (19, 20). Flow cytometry analysis using an anti-ACE2 antibody revealed that the cell surface expression levels of ACE2 orthologs from different species were comparable (Fig. S2A and B). All the tested Omicron variants could effectively infect cell lines expressing ACE2 from diverse species, albeit with significant variations in infection efficiency (Fig. 2A and B). Specifically, the XEC.25.1 and NB.1.8.1 variants exhibited enhanced infectivity relative to the JN.1 strain in the majority of cell lines, but their infectious capacity was attenuated in cells expressing mouse, rat, and ferret ACE2. The KP.3, LP.8.1.1, and XFG variants exhibited lower infectivity than the JN.1 strain in most cell types. Overall, Omicron subvariants exhibited significantly higher infection susceptibility toward cells expressing human, camel, and pig ACE2 compared to ferret and rat models. It is worth noting that pseudoviruses carrying the spikes of D614G and LP.8.1.1 have relatively weak infection efficiency in cells expressing ACE2 derived from mice, rats, and ferrets. Collectively, these findings demonstrate that JN.1 descendant variants exhibit broad potential for cross-species transmission. Certain emerging viral lineages exhibit enhanced cross-species infectivity due to structural remodeling, likely driven by selective pressure for immune evasion in the host.

### JN.1 descendant variants exhibited attenuated receptor-binding affinity except for the NB.1.8.1 strain

**Fig 3:**
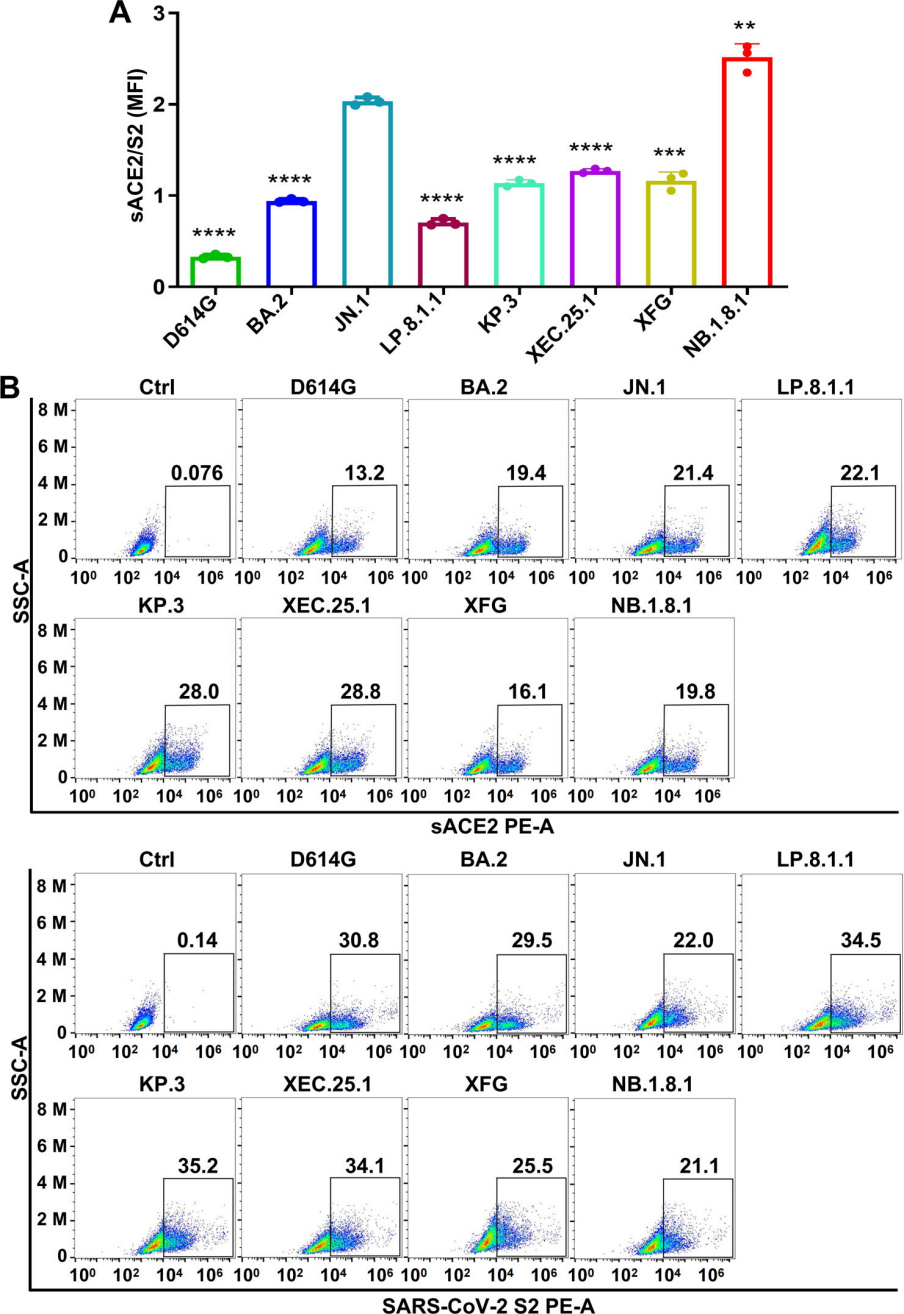
Evaluation of SARS-CoV-2 spike-binding affinity for soluble ACE2. (**A**) Comparison of binding affinity between spike proteins of emerging JN.1 subvariants and soluble ACE2. Binding affinity was measured by mean fluorescence intensity (MFI), normalized to spike expression levels (sACE2/S2), and weighted according to the proportion of positive cells. All statistical analyses were performed with the JN.1 variant as the reference. The experiments were performed with three technical replicates and repeated at least twice. Data from one representative experiment were presented as the mean ± SD. Statistical analysis utilized two-tailed Student’s *t*-tests, and *P* values are indicated as *P  <  0.05, **P  <  0.01, ***P  <  0.001, ****P  <  0.0001. (**B**) Flow cytometry plots of the binding affinity between the JN.1 subvariant spike and soluble ACE2. 293T cells expressing the variant spikes were incubated with Fc-tagged recombinant soluble human ACE2. Fc-tagged proteins were detected using a PE-conjugated anti-human IgG Fc secondary antibody.

To evaluate the receptor-binding capacity of the variant spikes, we measured their interaction with soluble recombinant human ACE2 using flow cytometry. Consistent with previous reports, the Omicron variants' spike exhibit significantly stronger binding affinities for the ACE2 receptor compared to the ancestral D614G strain (Fig. 3A and B). Among them, the NB.1.8.1 variant exhibited superior receptor-binding capacity compared to the JN.1 strain. Its binding efficiency was 7.6 times higher than that of D614G and 1.2 times higher than that of JN.1. Nevertheless, several other JN.1 sublineages, including KP.3, LP.8.1.1, XEC.25.1, and XFG, showed reduced receptor-binding efficiency. Notably, the LP.8.1.1 variant exhibited the most severe impairment in ACE2-binding affinity among these variants, with a 2.9-fold reduction in binding efficiency relative to JN.1. The attenuated receptor-binding capacity may be linked to the reduced cellular infectivity observed in certain variants.

### Enhanced membrane fusion activity of the XEC.25.1 spike

**Fig 4:**
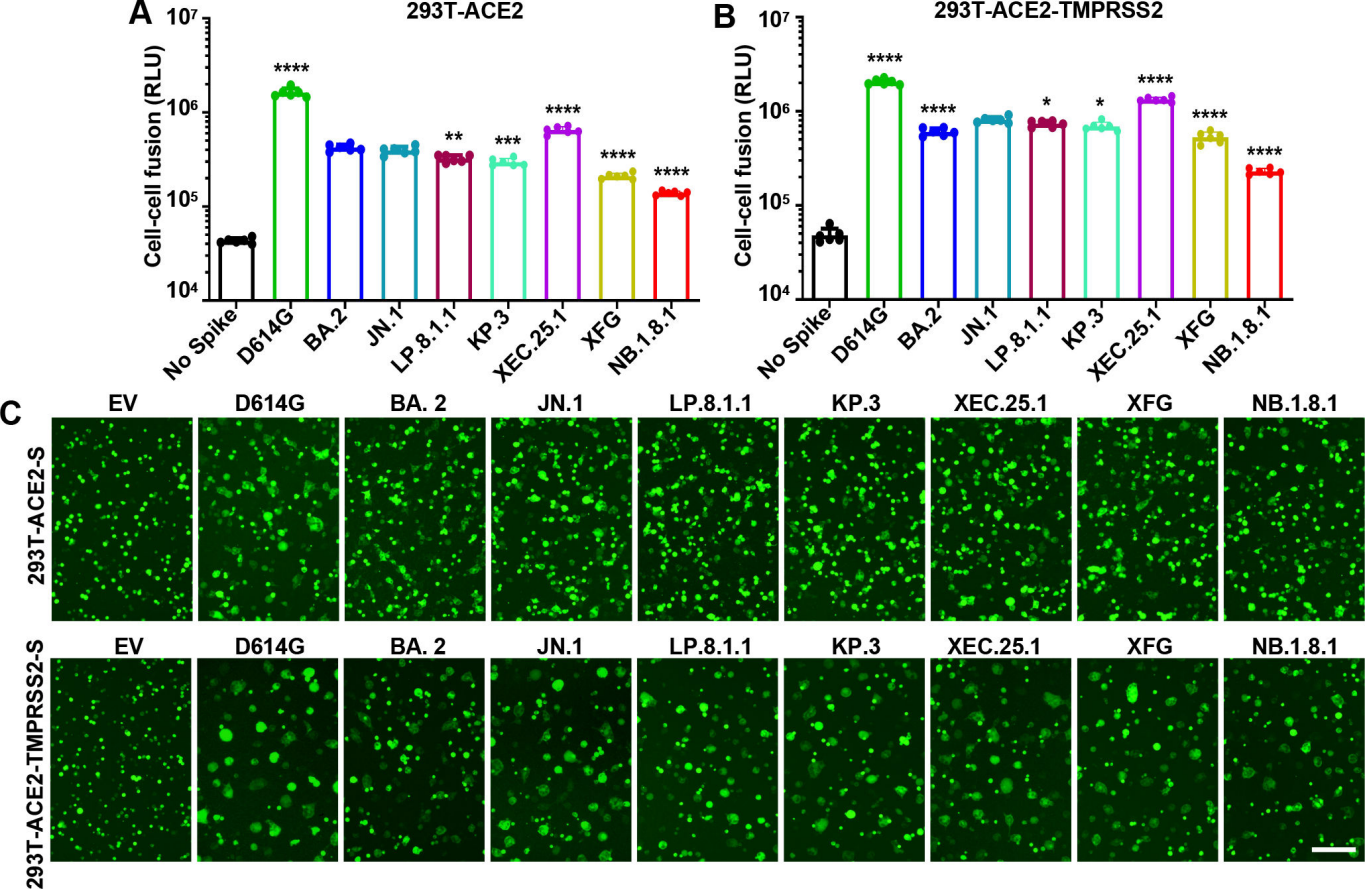
Fusogenicity mediated by SARS-CoV-2 variant spikes in 293T-ACE2 or 293T-ACE2-TMPRSS2 cells. (**A and B**) Cell–cell fusion mediated by SARS-CoV-2 variant spike proteins in 293T-ACE2 (A) or 293T-ACE2-TMPRSS2 (B) cells were determined using a T7 polymerase reporter system. All statistical analyses were conducted in comparison to the JN.1 variant. The experiments were performed with six technical replicates and repeated at least twice. Data from one representative experiment were presented as the mean ± SD. Statistical significance was determined by two-tailed Student’s *t*-tests, with significance indicated by *P < 0.05, **P < 0.01, ***P < 0.001, and ****P < 0.0001. EV represents the empty vector. (**C**) Representative images of cell fusion activity induced by ACE2 and SARS-CoV-2 variant spikes (scale bar = 400 µm).

To investigate the membrane fusion properties of emerging SARS-CoV-2 variants, we evaluated the fusogenic activity mediated by their spikes in target cells. JN.1 and its descendant variants displayed diminished syncytium formation capability relative to the ancestral D614G strain. Compared to JN.1, the LP.8.1.1, KP.3, XFG, and NB.1.8.1 variants exhibited a reduction in membrane fusion capacity (Fig. 4A through C). Notably, the XEC.25.1 variant exhibited enhanced cell-cell fusion activity relative to JN.1, and this effect was particularly pronounced in the 293T-ACE2-TMPRSS2 co-culture system. These findings suggest that currently circulating variants have diverged in their membrane fusion properties, with XEC.25.1 exhibiting superior fusogenicity that may contribute to its enhanced cellular infectivity.

### The NB.1.8.1 variant preferentially enters host cells via the endocytic pathway

**Fig 5:**
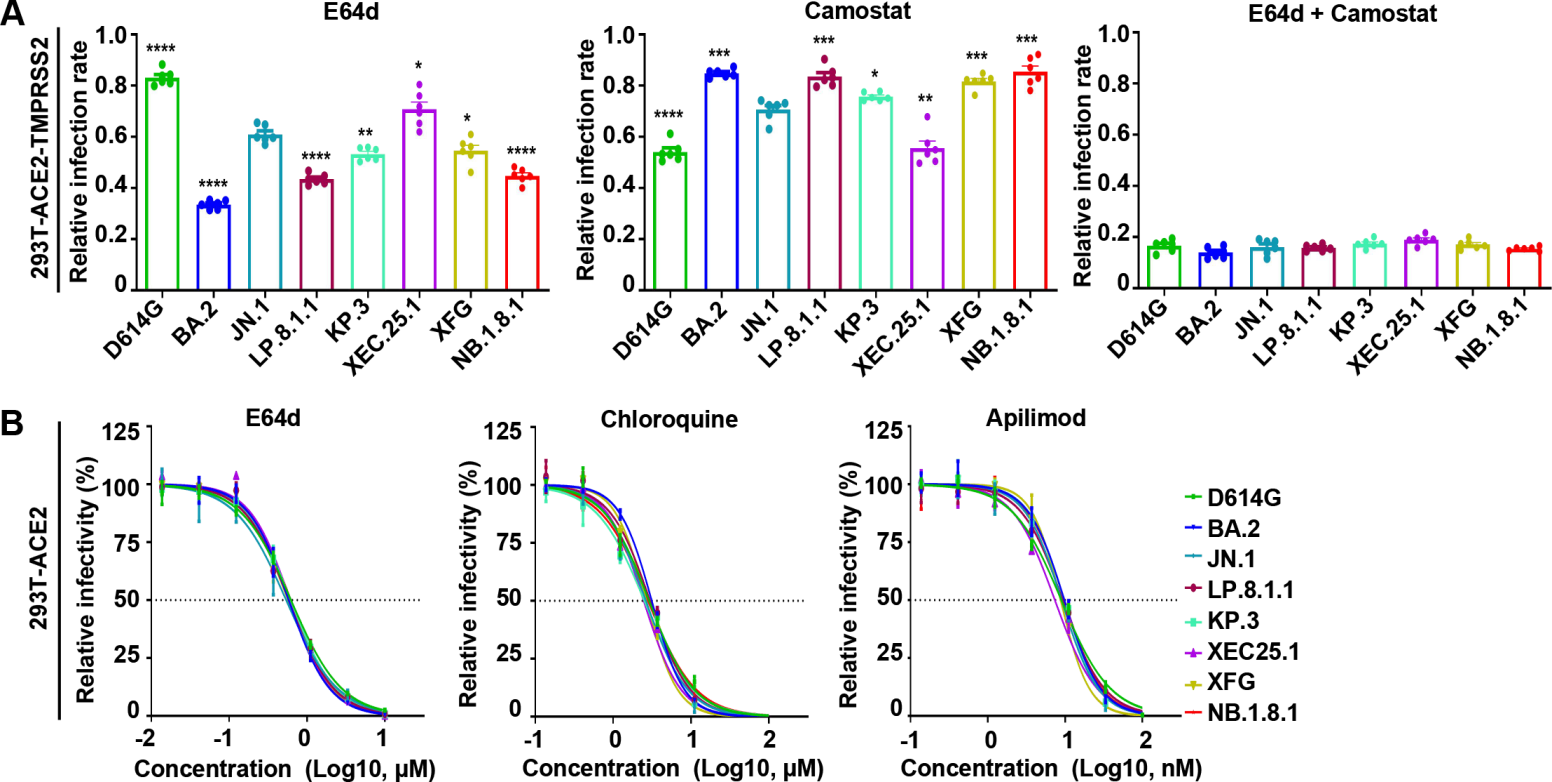
Inhibition of variant pseudovirus entry into 293T-ACE2 or 293T-ACE2-TMPRSS2 cells by E64d, Camostat, Chloroquine, and Apilimod. (**A**) Relative infectivity of SARS-CoV-2 variant pseudoviruses in 293T-ACE2-TMPRSS2 cells pretreated with E64d (5 μM) and/or Camostat (50 μM). Asterisks denote statistically significant difference compared to the JN.1 variant. The experiments were performed with six technical replicates and repeated at least twice. Data from one representative experiment were presented as the mean ± SD. Statistical significance was determined by two-tailed Student’s *t*-tests, with significance indicated by *P < 0.05, **P < 0.01, ***P < 0.001, and ****P < 0.0001. (**B**) 293T-ACE2 cells were pretreated with different concentrations of inhibitors (E64d, Chloroquine, and Apilimod) and then infected with variant pseudoviruses. The experiments were performed with three technical replicates and repeated at least twice. Data from one representative experiment were presented as the mean ± SD.

SARS-CoV-2 binds to the host ACE2 receptor and fully activates the spike protein via TMPRSS2 on the cell surface or cathepsins in endosomes, thereby mediating viral entry into target cells (21). To investigate the cellular entry mechanisms of currently circulating variants, we characterized the pathway tropism of viral entry in 293T-ACE2-TMPRSS2 cells. Prior to infection with variant pseudoviruses, cells were pretreated with either the TMPRSS2 inhibitor Camostat or the cathepsin inhibitor E64d. As shown in Fig. 5A, the SARS-CoV-2 D614G variant primarily utilized TMPRSS2 for host cell entry, whereas most JN.1 descendant strains exhibited a stronger reliance on the endocytic pathway. Compared to JN.1, the cellular entry of LP.8.1.1, KP.3, XFG, and NB.1.8.1 was primarily inhibited by E64d, indicating their preferential utilization of the endosomal pathway for host cell infection. In contrast, the XEC.25.1 variant exhibited a distinct entry tropism divergent from other strains, demonstrating a preferential dependence on the TMPRSS2-mediated membrane fusion pathway. To further verify the effect of virus through the endocytic pathway, we assessed the inhibitory effect of three endocytosis inhibitors, E64d, Chloroquine, and Apilimod, in 293T-ACE2 cells. All tested inhibitors exhibited significant inhibitory activity against JN.1 subvariants, with largely consistent efficacy profiles observed across different viral strains (Fig. 5B). These results indicate that in 293T-ACE2 cells, which lack endogenous TMPRSS2 expression, SARS-CoV-2 infection occurs primarily via the endosomal pathway. Together, our data suggest that inhibitors targeting the viral entry pathway hold therapeutic potential against JN.1 subvariants.

### JN.1 descendant variants exhibit enhanced immune evasion capabilities

**Fig 6:**
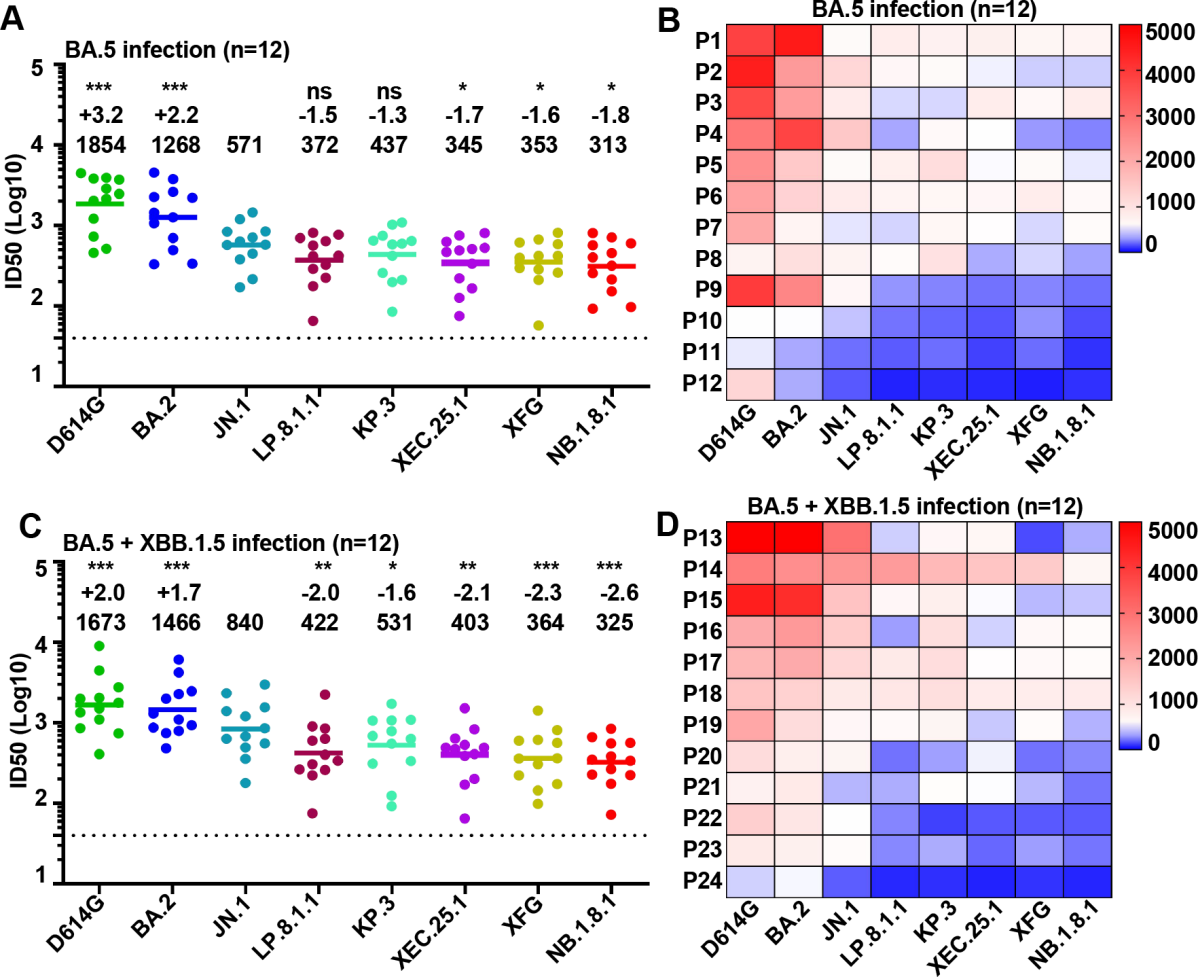
Neutralizing activity of human sera against emerging JN.1-related variants. (**A and B**) Neutralizing antibody titers in convalescent sera from individuals with BA.5 breakthrough infection were measured against JN.1-related variants. (**C and D**) Neutralizing antibody titers in convalescent sera from individuals with BA.5 + XBB.1.5 breakthrough infection were measured against JN.1-related variants. Heat maps (**B and D**) depict ID50 titers for each individual serum sample tested against SARS-CoV-2 variants. The geometric mean titer (GMT) for each variant is displayed above its respective data point. Fold-change differences in ID50 titers between variants and JN.1 are annotated above the GMT values. *P* values represent comparisons of all SARS-CoV-2 subvariants relative to the JN.1 variant. Statistical analysis was conducted using the Wilcoxon paired signed-rank tests. *P* values are displayed as *P  <  0.05, **P  <  0.01, ***P  <  0.001, ****P  <  0.0001; ns, not significant. Dashed lines represent the initial dilutions of serum samples.

Mutations in the SARS-CoV-2 spike may facilitate immune evasion, thereby compromising the efficacy of neutralizing antibodies induced by vaccination or prior infection. To investigate the antigenic evolution of recently circulating variants, we evaluated the neutralizing activity of convalescent sera from COVID-19 recovered individuals against SARS-CoV-2 variants. Two types of convalescent serum samples were analyzed, including sera from individuals recovered from BA.5 infection and sera from those with sequential BA.5 and XBB.1.5 infections. Both serum samples exhibited potent neutralizing activity against the ancestral D614G and BA.2 variants (Fig. 6A through D; Fig. S4A and B). Neutralizing antibody activity was significantly reduced in all JN.1-related subvariants compared to D614G and BA.2. It is noteworthy that JN.1 descendant variants exhibit significantly reduced neutralizing antibody activity compared to the ancestral JN.1 strain. Among participants with BA.5 infection, the neutralizing antibody ID50 titers against LP.8.1.1, XEC.25.1, XFG, and NB.1.8.1 decreased by 1.5-, 1.7-, 1.6-, and 1.8-fold, respectively (Fig. 6A and B; Fig. S4A). In participants with BA.5 + XBB.1.5 breakthrough infections, the corresponding ID50 titers declined by 2.0-, 2.1-, 2.3-, and 2.6-fold, respectively (Fig. 6C and D; Fig. S4B). However, the JN.1 descendant variants LP.8.1.1, XEC.25.1, XFG, and NB.1.8.1 exhibited comparable immune escape capabilities. The markedly attenuated serum neutralizing activity against emerging JN.1 descendant variants may serve as a pivotal factor in reinfection mediated by these novel viral strains.

### JN.1-related variants exhibit reduced susceptibility to neutralizing monoclonal antibodies

**Fig 7:**
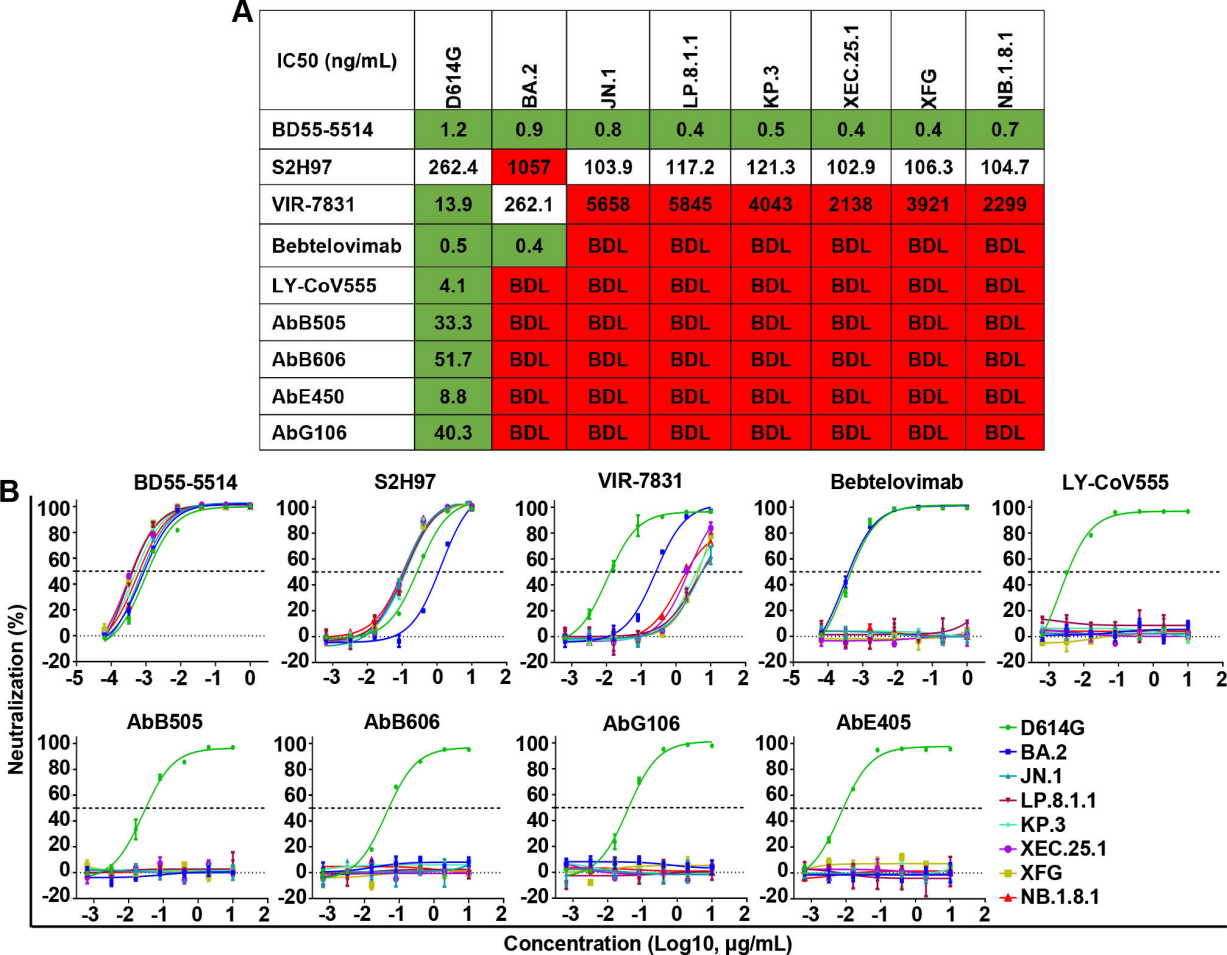
Neutralizing activity of mAbs against SARS-CoV-2 variant pseudovirus. (**A**) Neutralizing activity of mAbs against SARS-CoV-2 variant pseudovirus. Green, IC50 < 100 ng/mL; Red, IC50 > 1,000 ng/mL; BDL, below the detection limit. (**B**) Neutralization of SARS-CoV-2 variant pseudoviruses by diluted mAbs. Neutralizing activity was defined as the percent reduction in luciferase activity relative to the virus control group. The experiments were performed with two technical replicates.

The widespread therapeutic use of recombinant mAbs against COVID-19 is being challenged by Omicron spike mutations, which substantially enhance viral immune evasion. We evaluated the neutralization susceptibility of JN.1-related variants to various therapeutic mAbs. The results revealed a significant reduction or complete loss of neutralizing efficacy for most mAbs against JN.1-related variants (Fig. 7A and B). While VIR-7831 retained partial neutralizing activity against BA.2, its efficacy was markedly reduced against JN.1-related variants. Notably, mAb BD55 exhibited potent neutralization across all SARS-CoV-2 variants. Additionally, the mAb S2H97 exhibited broad neutralizing capability against all tested SARS-CoV-2 variants, showing superior neutralization against JN.1-related variants compared to BA.2.

## DISCUSSION

The continuous emergence of novel SARS-CoV-2 variants poses a great challenge to controlling the COVID-19 pandemic. Viral infectivity and immune evasion represent two critical risk characteristics of currently emerging SARS-CoV-2 variants (15, 21–23). The accelerated spread of new variants suggests that they may have acquired enhanced infectivity. For instance, in the early stage of the COVID-19 epidemic, the D614G strain rapidly replaced the original strain and became the dominant variant, primarily due to enhanced viral replication and infectivity conferred by the spike D614G mutation (24, 25). BA.2.86 exhibited low fusogenicity and infectivity in 293T-ACE2 cells. However, in cells with high TMPRSS2 expression, such as Calu-3 cells, it demonstrated markedly elevated fusogenicity and infectivity (2). In this study, we investigated the effects of the JN.1 subvariants on viral infectivity, antigenicity, and membrane fusion activity. Our findings indicate that most circulating variants exhibit significantly altered infectivity in mammalian cells relative to the ancestral JN.1 strain. The LP.8.1.1, KP.3, and XFG variants exhibited diminished infectivity, cell-cell fusion, and receptor-binding affinity relative to JN.1. Spike protein mutations in JN.1 subvariants are key factors that alter viral infectivity. The V1104L mutation may reduce the transition efficiency of the spike from the pre-fusion to the post-fusion state, thereby impairing viral infectivity (26). The L455S and F456L mutations diminish hydrophobic interactions within the RBD, leading to reduced ACE2-binding affinity and compromised viral infectivity (26). Compared to the JN.1 variant, the XEC.25.1 and NB.1.8.1 variants harbor additional T22N, F59S, and A435S mutations in their spikes. The F59S mutation, in particular, plays a critical role in increasing viral infectivity in susceptible cells, thereby potentially augmenting the target cell infection capacity of the XEC.25.1 and NB.1.8.1 variants (27).

The infectivity of SARS-CoV-2 is primarily determined by the binding affinity of its spike for the ACE2 receptor, which is affected by multiple factors. While most novel spike mutations impair ACE2 binding, a subset are well-tolerated and may even enhance receptor-binding affinity. The SARS-CoV-2 N501Y mutation enhances the affinity of spikes to ACE2, thereby contributing to increased viral infectivity (28). However, the receptor-binding affinity of the SARS-CoV-2 spike is impaired by the K417N mutation (29, 30). Consistent with previous reports, we demonstrated that the spikes of the JN.1 variant and its descendants exhibit higher ACE2-binding affinity than the D614G strain. We observed that the NB.1.8.1 variant demonstrates superior receptor-binding capability relative to JN.1, a characteristic that may underlie its increased infectivity in diverse cellular models. Studies have shown that the Q493E mutation alone reduces the binding affinity to ACE2. However, in the presence of F456L, the Q493E mutation enhances the binding affinity to ACE2 (31). The SARS-CoV-2 F59S mutation disrupts the hydrophobic interaction between F59 and its adjacent residues, increasing the flexibility of the spike and enhancing its adaptability for binding to the receptor (27). Moreover, NB.1.8.1 harbors several newly emerged mutations, including A435S and T478I. Its enhanced ACE2-binding affinity likely results from the synergistic effects of multiple spike mutations. Surprisingly, most emerging JN.1 subvariants, particularly KP.8.1.1, demonstrate reduced spike-ACE2-binding affinity compared to the parental JN.1 strain, which correlates with decreased cellular infectivity. Interestingly, XEC.25.1 exhibited reduced affinity to ACE2 compared to JN.1, yet demonstrated significantly higher infectivity in some cells. We speculate that this divergence may be attributed to novel mutations in the XEC.25.1 spike that enhance proteolytic processing efficiency. The underlying mechanism warrants further investigation.

SARS-CoV-2 entry requires activation of the spike by target cell proteases, which triggers proteolytic cleavage at the S1/S2 and S2′ sites and subsequently mediates viral fusion with the target cell membrane (12, 21). The Delta variant harbors the P681R mutation, which is suggested to enhance spike cleavage and promote fusogenic activity, thus potentially explaining its augmented infectivity in TMPRSS2-expressing cells (32, 33). In contrast, early Omicron variants carried the P681H mutation, which co-occurred with H655Y and N679K near the furin cleavage site. These mutations collectively reduced S1/S2 cleavage efficiency, thereby impairing membrane fusion (8, 34, 35). Consequently, the virus became less dependent on TMPRSS2 for cellular entry and more reliant on the endocytic pathway. Our study shows that JN.1 and its related variants exhibit significantly reduced spike-mediated membrane fusion compared to the D614G variant. It was surprising that most JN.1 subvariants, including LP.8.1.1, KP.3, XFG, and NB.1.8.1, exhibited weaker fusogenic activity than the parental JN.1 strain. JN.1 subvariants have acquired numerous novel mutations in the spike that may influence their membrane fusion activity. Studies have reported that the antibody evasion conferred by the F456L mutation comes at the cost of impaired spike-mediated membrane fusion (26). In addition, the S31 deletion in the LP.8.1.1 variant may attenuate fusogenicity by impairing spike binding to the ACE2 receptor (36). The N-terminal domain (NTD) of SARS-CoV-2 plays a key role in maintaining the conformation of the spike protein. The T22N mutation introduces an N-linked glycosylation site that may generate steric hindrance, thereby restricting the conformational flexibility required for efficient fusion (27). In the case of G184S, the substitution of glycine with serine enables hydrogen bond formation, potentially enhancing interactions within the NTD or with adjacent domains, which, in turn, reduces cell fusion activity (37). Collectively, the T22N and G184S mutations in NB.1.8.1 may coordinately impair spike-mediated membrane fusion. However, the XEC.25.1 variant exhibited enhanced membrane fusion activity compared to the JN.1 strain. This biological discrepancy may be attributed to specific mutations in its spike, namely, the additional F59S and A435S mutations relative to KP.3, which may act synergistically to potentially confer increased fusogenicity and viral infectivity.

Previous studies have confirmed that SARS-CoV-2 possesses zoonotic transmission potential, which primarily stems from the binding mechanism between the viral spike and host receptors (19, 38, 39). Due to the high conservation of the ACE2 receptor across various mammalian species, SARS-CoV-2 exhibits broad potential for cross-species infection. Studies have confirmed that SARS-CoV-2 can establish efficient transmission chains within animal populations such as minks, cats, and dogs (38, 39). This reservoir establishment drives viral evolution and poses a persistent and severe threat to global public health security. Our findings demonstrated that the NB.1.8.1 and XEC.25.1 variants confer enhanced viral infectivity in cells expressing ACE2 from various animal species, with a more pronounced effect observed in those with low susceptibility to the original virus. However, similar to the early D614G strain, the LP.8.1.1 variant exhibited limited infectivity in mouse, rat, and ferret models. These findings suggest that emerging SARS-CoV-2 variants may alter virus transmission chains via animal hosts. Zhang et al. evaluated the susceptibility of ACE2 orthologs from various species and found that cells expressing pig or cat ACE2 were highly sensitive to SARS-CoV-2, which aligns with our findings (39). Although pig ACE2 can mediate viral entry *in vitro*, animal studies indicate that pigs are not susceptible to the virus under natural conditions (40–42). This discrepancy is primarily attributed to the extremely low expression levels of endogenous ACE2 in the porcine respiratory tract (43). The transient transfection system used in this study leads to a high density of ACE2 on the cell surface. This non-physiological overexpression far exceeds the levels found in porcine respiratory tissues, potentially lowering the threshold required for viral entry.

The continuous evolution of SARS-CoV-2 during transmission underscores the formidable challenges to current epidemic containment efforts. Consistent with predictions, the emerging JN.1 subvariants LP.8.1.1, XEC.25.1, XFG, and NB.1.8.1 maintain potent immune evasion properties, which likely contribute to the recent increase in COVID-19 cases. Although individuals can generate specific neutralizing antibodies following Omicron variant infection, research indicates that the cross-neutralizing potency of these antibodies against newly emerging variants is substantially diminished. Specifically, compared to the D614G and BA.2 variants, all JN.1-related variants showed a significant reduction in neutralizing antibody titers, which is consistent with previous observations. Notably, several JN.1 subvariants, such as LP.8.1.1, XEC.25.1, XFG, and NB.1.8.1, exhibited a further reduction in neutralizing antibody levels. This phenomenon may originate from the accumulation of novel mutations in the spike of these new variants, leading to more pronounced antigenic evolution and consequently enabling them to successfully evade the immune protection established by prior infection (4, 44). Pseudovirus neutralization assays revealed that JN.1 and its derived lineages exhibited markedly increased resistance to neutralization by mAbs compared to the Omicron BA.2 variant. Studies have shown that the XFG variant exhibits significant resistance to class 1 antibodies, primarily due to the N487D and Q493E mutations (4). Furthermore, the XFG spike N487D substitution confers resistance to Class 1/2 neutralizing antibodies. Likewise, the T478I mutation in NB.1.8.1 confers enhanced escape capability against class 1/2 antibodies. Notably, the A435S mutation in XEC.25.1 and NB.1.8.1 confers broad resistance by reducing antibody neutralization across all epitopes. These findings imply that pre-existing immunity from early viral strains may not confer robust protection against reinfection caused by these emerging variants. The observation that antibodies induced by vaccination or prior infection exhibit only partial neutralization against emerging SARS-CoV-2 variants underscores the imperative to develop next-generation vaccines effective against both currently circulating strains and potential future variants.

In conclusion, this study systematically characterized the biological properties of JN.1 descendant variants, showing that specific strains, including XEC.25.1 and NB.1.8.1, exhibit significant evolutionary advantages over the parental JN.1 strain, with a markedly enhanced capacity to infect susceptible cells. Furthermore, the emerging JN.1 sublineages exhibit increased resistance to neutralization by convalescent sera from Omicron infections, suggesting further antigenic drift and a partial ability to evade pre-existing immunity. Given the evolution of SARS-CoV-2 variants and their persistent threat to global public health, it is imperative to closely monitor the transmission dynamics of the JN.1 subvariants and accelerate the development of effective antibodies and vaccines.

## Data Availability

The data that support the findings of this study are available from the corresponding author upon reasonable request.

## SUPPLEMENTAL MATERIAL

Supplemental figures spectrum.03826-25-s0001.docx Fig. S1 to S4.

Table S1 spectrum.03826-25-s0002.xlsx Information of SARS-CoV-2 convalescent patients participating in this study.

ASM does not own the copyrights to Supplemental Material that may be linked to, or accessed through, an article. The authors have granted ASM a non-exclusive, world-wide license to publish the Supplemental Material files. Please contact the corresponding author directly for reuse.
