# Supplementary material for: Immune evasion, infectivity, and membrane fusion of SARS-CoV-2 variants LP.8.1.1, XEC.25.1, XFG, and NB.1.8.1
Source: Microbiol Spectr. 2026 Jun 24;14(8):e03826-25. doi: 10.1128/spectrum.03826-25 (PMC13436279; doi:10.1128/spectrum.03826-25)
Supplement: Supplementary file 1 [file spectrum.03826-25-s0001.docx]

**Supplementary Figures**

**Figure S1 Characterization of the major currently circulating SARS-CoV-2 variant.**

**A** Schematic of the SARS-CoV-2 spike protein structure.

**B** Amino acid mutation profile of SARS-CoV-2 variant spike proteins.

**Figure S2 Expression levels of ACE2 homologues from different species in 293T cells, related to Figure 2.**

A The expression levels of ACE2 of different species in 293T cells were analyzed by flow cytometry (red fluorescence activated by H2BmRuby3).

B Flow cytometric analysis of ACE2 expression levels in 293T cells across different species. Cells expressing ACE2 were incubated with rabbit anti-ACE2 polyclonal antibody (Sino Biological Inc., Cat: 10108-T24). After washing, cells were stained with 2 μg/mL Alexa Fluor 488-conjugated goat anti-rabbit IgG (H+L) and subsequently analyzed by flow cytometry.

**Figure S3 Flow cytometry analysis of the binding between SARS-CoV-2 subvariant spike proteins and soluble ACE2, related to Figure 3.**

A The BA.2 and JN.1 variant spike proteins bind to different concentrations of soluble ACE2. The experiments were performed with 2 technical replicates and repeated at least twice. Data from one representative experiment were presented as the mean ± SD.

B Flow cytometry plots showing the binding between SARS-CoV-2 subvariant spike proteins and soluble ACE2.

**Figure S4 Neutralizing activity of human serum against emerging JN.1-related variants, related to Figure 6.**

Neutralization curves against various SARS-CoV-2 variants are shown for convalescent sera from participants after BA.5 infection (A) or from participants after BA.5 + XBB.1.5 breakthrough infections (B). Neutralizing activity was determined as the percentage reduction in luciferase activity relative to the virus control group.
